# Frosted branch angiitis in a patient with systemic juvenile idiopathic arthritis: a case report

**DOI:** 10.1186/s12886-024-03373-1

**Published:** 2024-03-05

**Authors:** Jarret L. Garbrecht, Zachary R. Powell, Cynthia K. McClard, Jila Noori

**Affiliations:** 1 University of Oklahoma College of Medicine, Oklahoma City, OK USA; 2 Department of Ophthalmology, Dean McGee Eye Institute, University of Oklahoma Health Sciences Center, 608 Stanton L Young Blvd, 73104 Oklahoma City, OK USA

**Keywords:** Systemic juvenile idiopathic arthritis, Frosted branch angiitis, Branch retinal vein occlusion, Optical coherence tomography, Fluorescein angiography, Case report

## Abstract

**Background:**

Frosted branch angiitis is a retinal vascular condition that is associated with a viral infection or autoimmune disorders like Crohn’s disease, systemic lupus erythematosus, and Behcet’s disease. Frosted branch angiitis presents with vascular inflammation, retinal edema, and severe retinal vascular sheathing. We present a case of systemic juvenile idiopathic arthritis, an autoinflammatory disease, presenting with frosted branch angiitis.

**Report of Case:**

A 14-year-old female with systemic juvenile idiopathic arthritis and a history of bilateral anterior uveitis developed acute unilateral vision loss and was found to have frosted branch angiitis complicated by branch retinal vein occlusion. She underwent an extensive serology workup and aqueous viral PCR to rule out other possible autoimmune and viral etiologies for forested branch angiitis. She received systemic and intravitreal antiviral treatment due to positive CMV IgM initially. However, the clinical picture improved following the use of a higher dose of oral steroids and the switch of the immunosuppressive agent to a TNF-a inhibitor.

**Conclusion:**

To our knowledge, this would be the first case in the literature demonstrating a systemic juvenile idiopathic arthritis patient presenting with frosted branch angiitis. Infectious causes still must be ruled out, especially CMV, as it is the most common cause of secondary frosted branch angiitis.

## Background

Frosted branch angiitis (FBA) is a rare disorder that has been associated with intraocular viral infection or with autoimmune disorders [1–5]. The majority of cases are reported in patients aged 2–42 years [3]. FBA presents with vascular inflammation, retinal edema, decreased visual acuity, and severe retinal vascular sheathing [1]. Inflamed retinal vessels exhibit a “frosted branch” appearance on the fundus exam. We report a case of FBA in a patient with systemic idiopathic juvenile arthritis (SJIA). Written informed consent has been obtained from the patient and family.

## Report of case

**Fig. 1:**
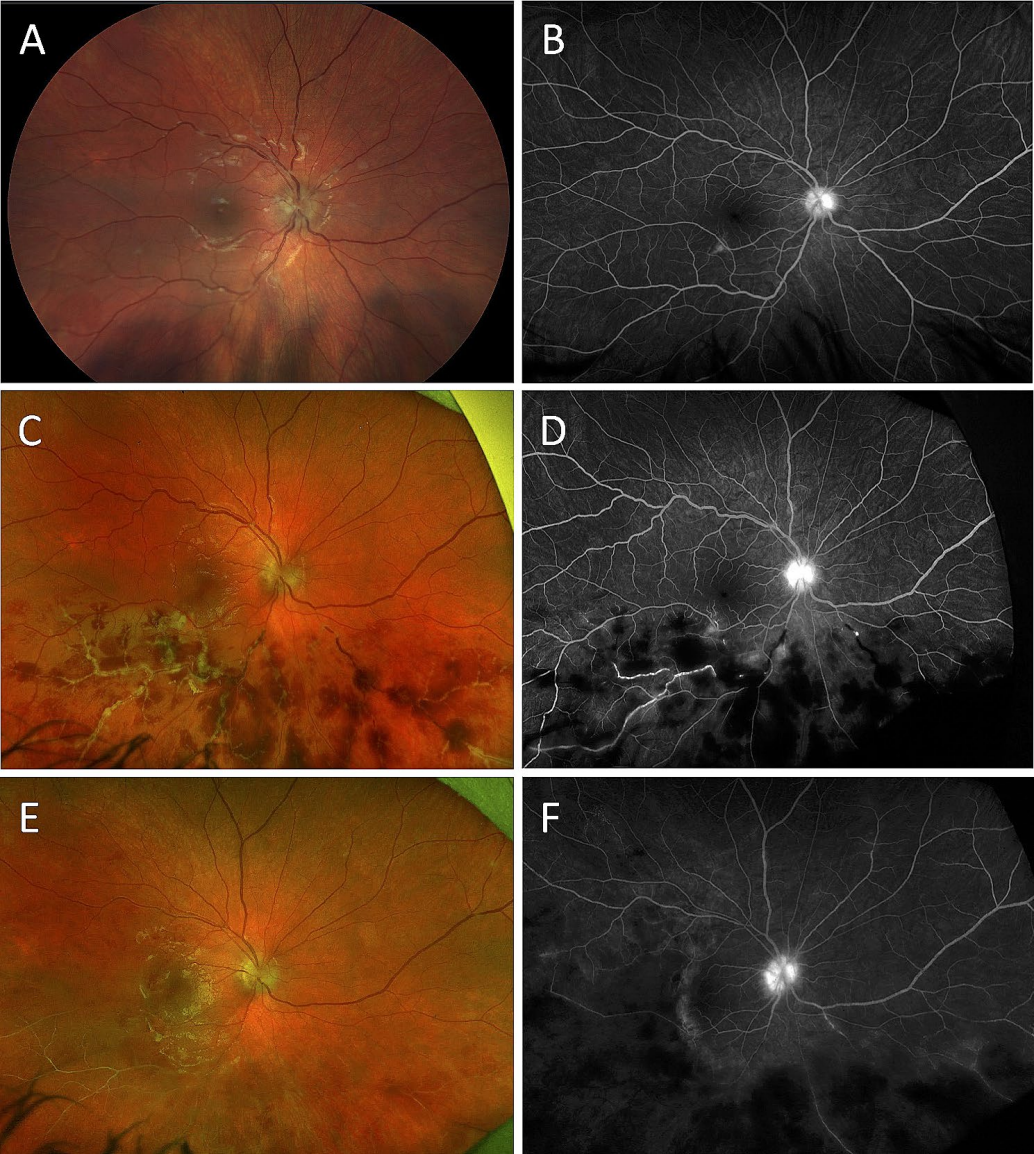
*A*, *B*, Initial evaluation of the right eye: color fundus photo (**A**) and fluorescein angiography at 5 min (**B**) demonstrating disc edema with leakage, perivenular sheathing, and vessel leakage. *C*, *D*, Development of frosted branch angiitis/vascular occlusion. Color fundus photo (**C**) and fluorescein angiography at 5 min (**D**) demonstrating inferior peripheral hemorrhages, persistent disc leakage, large vein segmentation with sheathing and leakage, and multiple focal areas of retinal ischemia. *E*, *F*, after treatment. Color fundus photo (**E**) and fluorescein angiography at 5 min (**F**) exhibiting residual retinal ischemia and sclerotic vasculature in the inferior macula

**Fig. 2:**
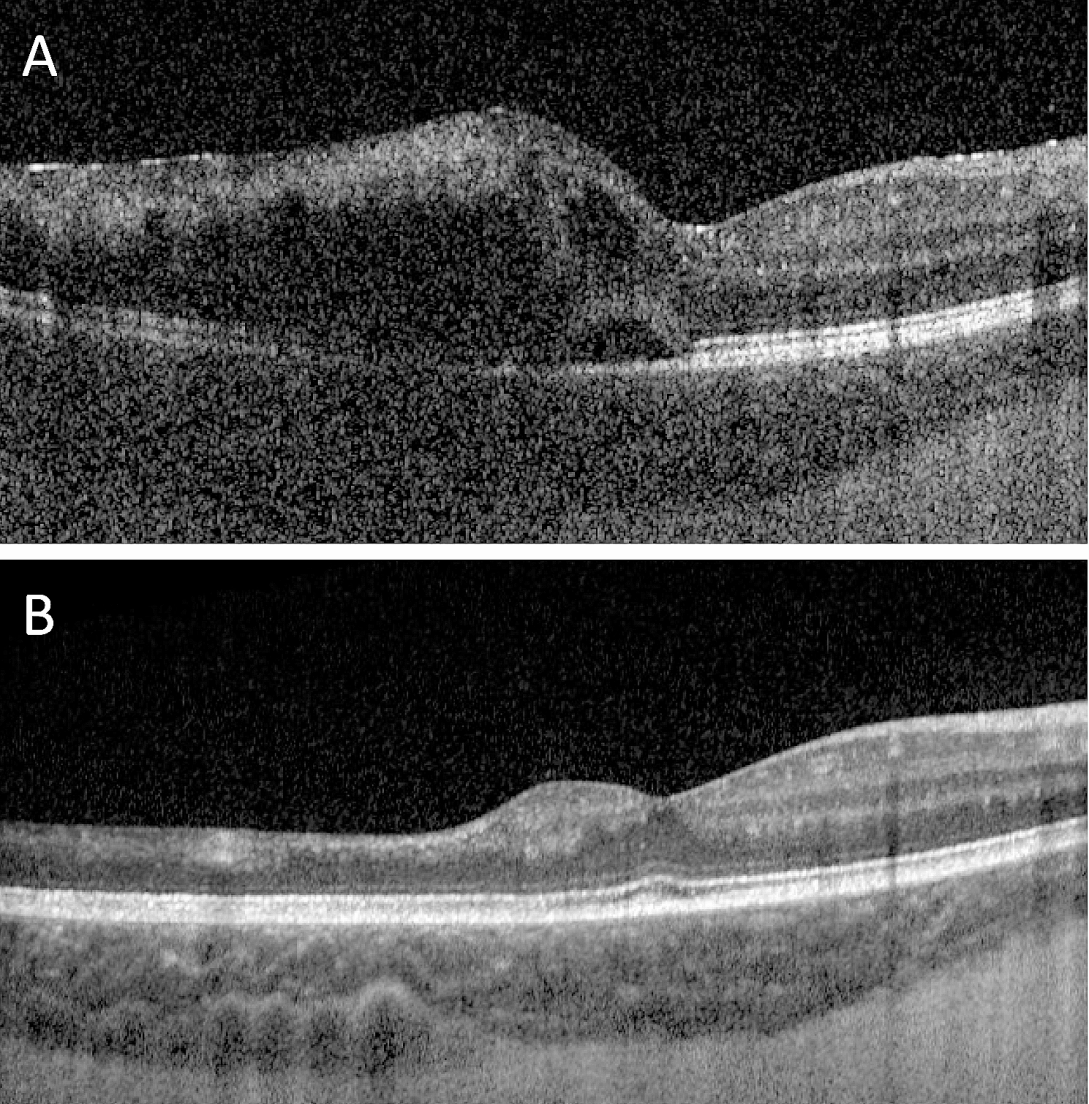
Optical coherence tomography of the macula of the right eye. **A**, Cystoid macular edema developed two months following the onset of frosted branch angiitis with occlusive vasculitis. **B**, Resolution of macular edema after three total injections of bevacizumab

A 14-year-old female with a history of SJIA on systemic steroids presented with bilateral anterior uveitis. The best corrected visual acuity was 20/25 and 20/20 in the right and left eyes, respectively, with normal eye pressures. The ocular exam was notable for 3 + anterior chamber cells in both eyes, bilateral keratic precipitates, and posterior synechiae in the left eye. The fundus exam was unremarkable except for two foci of peripheral perivascular pigmented chorioretinal scars in the left eye. The patient was treated with topical steroids and continued canakinumab injections for systemic inflammation, but she eventually developed inflammation in the vitreous and a few perivascular cotton wool spots in the retina. Fluorescein angiography (FA) of the fundus demonstrated leakage of the bilateral optic discs and vasculature (Fig. 1A, B). She was changed to tocilizumab systemically by her pediatric rheumatologist and was stable for four months when she developed acute vision loss with photopsia in the right eye. At this time, the best-corrected vision in the right eye was 20/125, with normal pressure. Fundus exam was notable for new retinal hemorrhages inferiorly involving the macula and new vascular sheathing (Fig. 1B). FA of the right eye revealed large vessel inflammation, segmentation in the retinal veins, and persistent disc edema (Fig. 1C, D), together representing unilateral FBA complicated by branched retinal vein occlusion (BRVO), with later onset of cystoid macular edema (CME) (Fig. 2A). Multiple QuantiFERON Gold tests were negative for tuberculosis (TB) during the course of her systemic illness and episodes of ocular inflammation. PCR tests of aqueous fluid were negative for cytomegalovirus (CMV), varicella zoster virus (VZV), herpes simplex virus (HSV) I/II, and Toxoplasma gondii. However, blood work revealed seropositivity for CMV IgM, EBV capsid antigen IgM and IgG, HSV I and II IgM, and HSV I IgG. She received 4 times intravitreal ganciclovir injections in conjunction with oral valganciclovir over 3 weeks without apparent improvement in the clinical exam. She was placed on daily suppressive valacyclovir later due to intolerance of valganciclovir. Meanwhile, her rheumatologist switched her immunosuppressive therapy from tocilizumab to infliximab, a TNF-α inhibitor, and increased the dose of oral steroid from 5 mg to 20 mg daily prednisone due to her active ocular and systemic findings, including arthritis and fever. At that time, fundus exams demonstrated perceivable gradual improvement in retinal hemorrhage and sheathing of the retinal vessels on follow-up visits. The patient was treated with intravitreal bevacizumab for macular edema three times, with a complete resolution of CME (Fig. 2B). At the four-month follow-up, the patient’s fundus exhibited improvement in hemorrhages and sheathing of vessels with multiple areas of capillary nonperfusion in the region of vessel occlusions (Fig. 1E, F). The patient demonstrated stabilization of systemic symptoms of SJIA on infliximab infusions, as well. Visual acuity at her most recent visit on 12 months follow-up was 20/25 in both eyes, and inflammation was bilaterally quiescent. Sectoral retinal laser photocoagulation was planned to prevent retinal neovascularization in her right eye.

## Discussion and conclusions

Juvenile idiopathic arthritis(JIA) is an inflammatory arthritis that occurs in children under 16 years old and lasts a minimum of six weeks. There are seven different subtypes of JIA with a common feature of chronic arthritis. Although the cause of JIA remains unclear, a commonality in pathophysiology consists of the activation of CD4 + helper T cells with an imbalance between Th1 and Th17 T cells. A subtype of JIA is systemic JIA (SJIA), which is characterized by a fever of at least 2-week duration and can be accompanied by other extra-articular features such as generalized lymphadenopathy, evanescent rash, splenomegaly, hepatomegaly, or serositis. However, the most common extra-articular manifestation is ocular inflammation [6]. 

Ocular inflammation seen in SJIA frequently presents as chronic anterior uveitis. Patients with chronic anterior uveitis are typically asymptomatic in the early stages; however, as the disease progresses, they can present with eye pain, eye redness, headache, and photophobia. Proper screening and urgent treatment are necessary to prevent further ocular complications such as glaucoma, cataracts, band keratopathy, and persistent cystoid macular edema that can potentially lead to visual impairment and blindness [7]. Although there are many ocular manifestations with SJIA patients, there has never been a link to FBA.

In a patient with a history of systemic inflammatory disease and on immunosuppressive therapy, FBA secondary to viral retinitis or from the underlying disorder must be considered. Viral PCR tests on aqueous fluid and serological studies can help evaluate etiology.

There are two classes of FBA: primary idiopathic and secondary, stemming from an underlying infection or disease. The mechanism of pathological tissue damage is hypothesized to be due to immune-complex deposition from a hypersensitivity reaction [3]. Viruses such as CMV, the most common cause of secondary FBA, are hypothesized to produce antigens that form immune complexes. Other infectious causes include HIV, EBV, HSV, VZV, T gondii, and TB [3, 5, 8–11]. Autoimmune diseases such as Crohn’s disease, systemic lupus erythematosus, and Behcet’s disease are other reported causes of secondary FBA [1, 4, 8, 12]. An FBA-like picture can also be seen in an atypical presentation for sympathetic ophthalmia [13]. 

Treatment of FBA involves antimicrobial therapy for infectious etiologies and anti-inflammatory agents for inflammatory etiologies [14, 15]. Loss of visual acuity secondary to FBA is often due to the development of macular edema [14]. Anti-VEGF therapy and laser treatment have been utilized to limit this complication [14, 16]. In our patient, serologies suggested multiple potential viral infections, and thus, treatment included multiple antiviral agents and routes. Despite negative aqueous PCR results, we decided to start antiviral treatment for our patient to prevent the progression of the retinal vascular inflammation and further loss of vision. However, we didn’t perceive a clinical improvement over time with this mode of treatment. Therefore, in conjunction with her pediatric rheumatologist decided to increase the dose of oral steroids and change her immunosuppressive treatment to a TNF-α inhibitor. The role of changes in the patient’s immunomodulatory therapy from canakinumab (an anti-IL-1β agent) to tocilizumab (an IL-6 inhibitor) is unclear in the pathogenesis of FBA in this case [15]. However, the patient’s systemic findings and ocular inflammation ultimately improved on infliximab infusion. We report this case to highlight that ocular inflammation in SJIA may present with FBA and to emphasize the importance of a thorough infectious and inflammatory workup in patients with this clinical presentation to guide timely management.

## Data Availability

Not applicable.

## Abbreviations

FBA: Frosted Branch Angiitis
SJIA: Systemic juvenile idiopathic arthritis
FA: Fluorescein Angiography
BRVO: Branch Retinal Vein Occlusion
CME: Cystoid Macular Edema
TB: Tuberculosis
CMV: Cytomegalovirus
VZV: Varicella Zoster Virus
HSV: Herpes Simplex Virus
EBV: Epstein-Barr Virus
JIA: Juvenile Idiopathic Arthritis
